# Mechanism of Restoration of Forelimb Motor Function after Cervical Spinal Cord Hemisection in Rats: Electrophysiological Verification

**DOI:** 10.1155/2017/7514681

**Published:** 2017-11-12

**Authors:** Takumi Takeuchi, Masahito Takahashi, Kazuhiko Satomi, Hideaki Ohne, Atsushi Hasegawa, Shunsuke Sato, Shoichi Ichimura

**Affiliations:** ^1^Department of Orthopaedic Surgery, Kyorin University, 6-20-2 Shinkawa, Mitaka-shi, Tokyo 181-0004, Japan; ^2^Department of Orthopaedic Surgery, Kugayama Hospital, 2-14-20 Kitakarasuyama, Setagaya-ku, Tokyo 157-0061, Japan

## Abstract

The objective of this study was to electrophysiologically assess the corticospinal tracts of adult rats and the recovery of motor function of their forelimbs after cervical cord hemisection. Of 39 adult rats used, compound muscle action potentials (CMAPs) of the forelimbs of 15 rats were evaluated, before they received left C5 segmental hemisection of the spinal cord, by stimulating the pyramid of the medulla oblongata on one side using an exciting microelectrode. All 15 rats exhibited contralateral electrical activity, but their CMAPs disappeared after hemisection. The remaining 24 rats received hemisection first, and CMAPs of 12 rats were assessed over time to study their recovery time. All of them exhibited electrical activity of the forelimbs in 4 weeks after surgery. The remaining 12 rats received additional right C2 segmental hemisection, and variation of CMAPs between before and after surgery was examined. The right side of the 12 rats that received the additional hemisection exhibited no electrical activity in response to the stimulation of the pyramids on both sides. These results suggest that changes in path between the resected and healthy sides, activation of the ventral corticospinal tracts, and propriospinal neurons were involved in the recovery of motor function after cervical cord injury.

## 1. Introduction

It is considered that a damaged central nervous system will never be restored [1], but we reported that the once paralyzed motor function of the forelimbs of juvenile rats had been restored due to a significant change that occurred in the corticospinal tract pathways after a brain injury [2]. We also assessed the motor function of the forelimbs of these rats following cervical cord hemisection and reported that restoration of motor function was observed in approximately 60% of the juvenile rats and 40% of the adult rats [3].

In past anatomical verification of experiments in corticospinal tract injury of rodents, it was reported that not only axonal re-elongation and collateral sprouting occurred in the damaged part but also the axons of intact neurons extended new branches in an area apart from the damage, thus allowing the hind limbs to recover better than the forelimbs [4–6]. However, this recovery process has never been electrophysiologically verified over time.

The objective of this research was to electrophysiologically assess the corticospinal tract of adult rats, then electrophysiologically verify the recovery of the forelimb motor function over time after cervical cord hemisection, and lastly, examine the compensation pathways for the recovery of motor function by conducting an additional hemisection.

## 2. Materials and Methods

### 2.1. Animals

Of 39 twelve-week-old male Wister rats used as experimental animals, 15 were used for the preliminary experiment prior to segmental hemisection of the cervical cord (group of rats for preliminary experiment), and the remaining 24 received cervical cord hemisection without being subjected to the preliminary experiment (group of rats for hemisection).

### 2.2. Surgical Procedure

Animals were first anesthetized by intraperitoneal administration of xylazine (10 mg/kg; Bayer HealthCare, Monheim, Germany) and ketamine (90 mg/kg; Daiichi Sankyo, Tokyo, Japan). For monitoring of vital signs during surgery, rectal temperature, arterial oxygen saturation, heart rate, and respiration rate were continuously measured.

**Figure 1:**
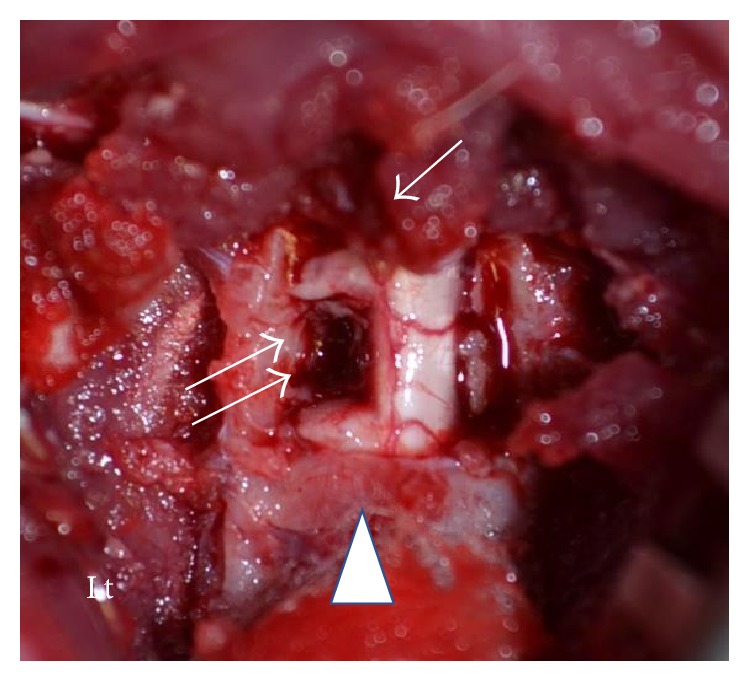
Macrographic image of the left C5 segmental hemisection of the cervical cord. Arrows: hemisected part; arrow: C2 spinous process; arrow head: C5 lamina; Lt: left.

The hemisection was performed as follows: the rat was fastened to a brain stereotaxic apparatus, the laminae were exposed by posterior approach between C2 and C5, and laminectomy was performed at C3 and C4. After dissecting the dura mater at that part, cervical cord hemisection at the left C5 segment was performed at a width of 2 mm in the head-to-tail direction, according to the method by coauthor Hasegawa, to prevent readhesion of nerves at the dissected part (Figure 1) [3]. The animals were administered subcutaneous (s.c.) injections of buprenorphine (0.02 mg/kg; Otsuka, Tokyo, Japan) at 12-hour intervals for 3 days as postoperative analgesia, in addition to intramuscular (i.m.) injection of penicillin G (22,000 units/kg; Tsumura & Co., Tokyo, Japan) once every 24 hours for 3 days as an antimicrobial prophylaxis.

**Figure 2:**
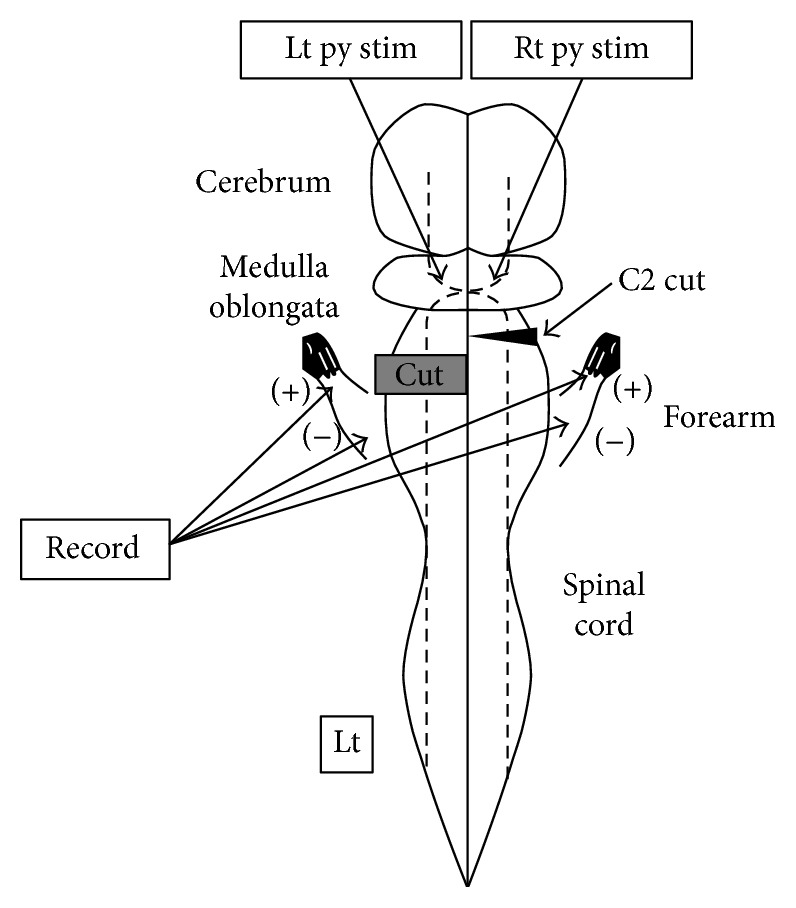
Left and right pyramidal tracts and measurement method. The left and right pyramids were stimulated separately, and CMAPs of both forelimbs were recorded. Py: pyramid; stim: stimulation; Lt: left; Rt: right; broken line: pyramidal tracts (plus sign in the medulla oblongata).

An additional hemisection was performed on 12 of the 24 hemisected rats, 6 weeks after the first hemisection. C1 and C2 laminae were exposed by posterior approach, a C2 laminectomy was performed, the dura mater at that part was dissected, and then a right C2 segmental cervical cord hemisection was performed (Figure 2).

### 2.3. Electrophysiological Procedures

For electrophysiological evaluation, the Neuropack MEB-2200® measuring system (Nihon Kohden, Tokyo, Japan), SS-203J® isolator (Nihon Kohden), and SEN-3401® microstimulator (Nihon Kohden) were used.

To expose the caudal brainstem, a posterior craniotomy was performed, and then an exciting electrode (TK212-048® epoxy-insulated microelectrodes; Unique Medical Co. Ltd., Tokyo, Japan) was installed on the left or the right pyramid of the medulla oblongata by inserting it at a position approximately 1.5 mm closer to the head and 0.5 mm toward the outside, and at an insertion angle of approximately 30° toward the head, with respect to the obex, and a constant current stimulation was provided, respectively [7, 8].

Bipolar recording was performed to examine the electric potential of the flexor and extensor muscles of the forelimbs using needle electrodes. Recording electrodes for the flexor and the extensor were, respectively, installed at positions away from the elbow at an interval of 10 mm to measure compound muscle action potentials (CMAPs) (Figure 2). The recording frequency band was set to fall within a range from 10 Hz to 3 kHz, and stimulation was provided 10 times in total under the following conditions: frequency, 1 Hz; ISI, 1 ms; duration, 0.2 ms; and trains, 3. For the stimulation intensity, the motion threshold which produces waveforms was regarded as 1 T, and the main experiment was performed at 2 T, where latency was stabilized.

### 2.4. Rats for Preliminary Experiment

**Figure 3:**
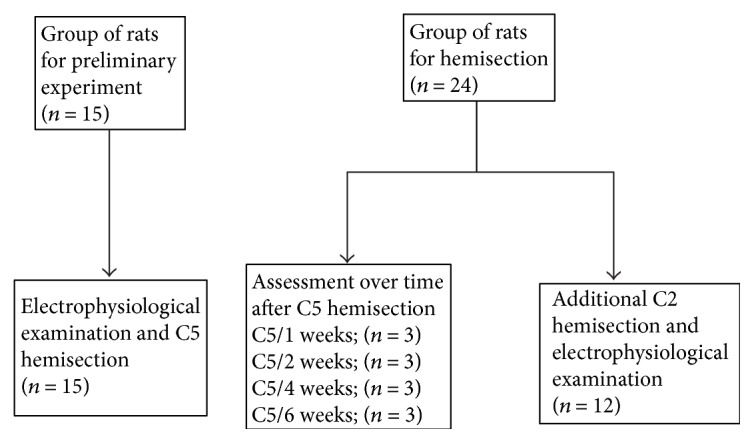
Experimental protocol. Group of rats for preliminary experiment: 15. Group of rats for hemisection: 24 (group assessed over time after C5 hemisection: 12; group that received additional C2 hemisection: 12).

Using the 15 rats for preliminary experiment, the motion threshold (1 T) was checked, and then the stimulation intensity was increased from 2 T to 8 T to check the effect of an increase on waveform forming on the stimulated side. The amplitude and latency were recorded at the optimum stimulation intensity, which allowed the latency to stabilize, and then a left C5 segmental hemisection of the cervical cord was performed (Figure 3).

### 2.5. Rats for Hemisection

Of the 24 rats that received a left C5 segmental hemisection without being subjected to the preliminary experiment, 12 were assessed over time to check the time of their functional recovery, with 3 each, respectively, assessed electrophysiologically at 1, 2, 4, and 6 weeks after the surgery (Figure 3).

The remaining 12 hemisected rats were subjected to an additional C2 segmental hemisection 6 weeks after the first hemisection. They received a right C2 segmental hemisection at the C2 lamina level, and electrophysiological evaluation was performed (Figure 3).

### 2.6. Evaluation of CMAPs

Peak-to-peak amplitudes were measured for both the flexor and extensor. For the latency, the duration from the first stimulation to the start of a waveform was measured.

### 2.7. Evaluation of Stimulated and Hemisected Areas

**Figure 4:**
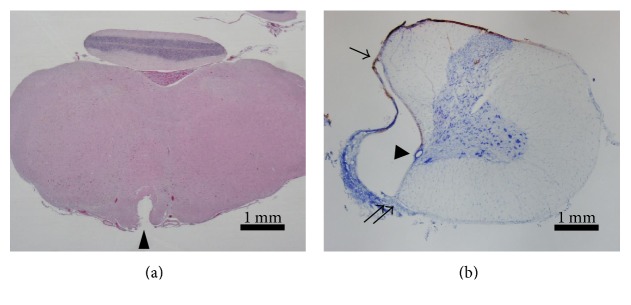
Pathological image of the stimulated part of the pyramid and the hemisected part of the cervical cord. (a) Microscopic image of the stimulated part of the pyramid having undergone burnout process (midbrain level); arrow head: pyramid. (b) Microscopic image of the hemisected part of the cervical cord (C5 segment level) (modification from document 3); arrow head: central canal; arrows: anterior median fissure; arrow: dorsal median sulcus.

To check the hemisected area of the cervical cord and the stimulated areas of the pyramid, the pyramids of all 39 rats having undergone electrophysiological assessment were burnt out at 20 mA, 50 Hz for 5 minutes. Then, pathological specimens were prepared as follows: under general anesthesia performed by administering 50 mg/kg of pentobarbital sodium into the abdomen, blood was removed using phosphate-buffered saline (PBS), and perfusion fixation was performed using a 4% paraformaldehyde buffer solution. The medulla oblongata and the cervical cord with a width of approximately 10 mm were cut out, with the stimulated area of the pyramid and the damaged area regarded as the center, and embedded by freezing using a 30% sucrose solution. The spinal cord was cut into thin films with a thickness of 20 *μ*m using a microtome, dyed using toluidine blue stain and hematoxylin and eosin stain, and observed under an optical microscope (Figures 4(a) and 4(b)).

### 2.8. Statistical Analysis

To analyze the CMAP amplitude and latency, three personnel performed the measurement twice, top and bottom outliers were excluded, and the average of the remaining values was used. Statistical analysis was conducted by performing the Mann–Whitney *U* test for the comparison of two groups and the Steel-Dwass test for the comparison of three or more groups, with the level of significance set to lower than 5%.

## 3. Results

### 3.1. Rats for Preliminary Experiment

**Figure 5:**
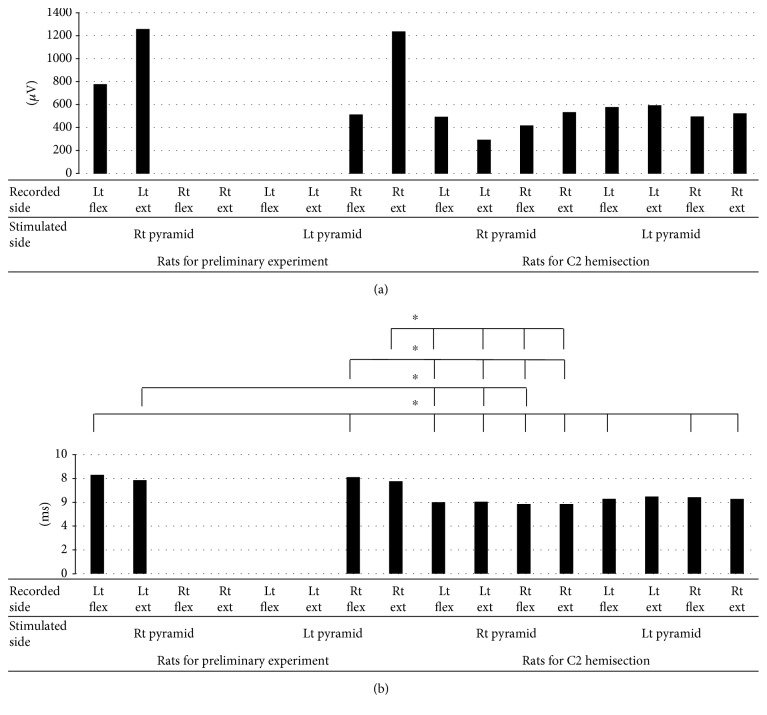
Amplitude (a) and latency (b) of CMAPs of the group of rats for preliminary experiment and the group of rats that received additional C2 hemisection. (a) No significant difference was found in the amplitude between the rats for preliminary experiment and the rats that received additional C2 hemisection. (b) Meanwhile, a significant shortening of latency was found in the rats that received C2 hemisection (^∗^*p* < 0.05). CMAPs: compound muscle action potentials; Rt: right; Lt: left; Flex: flexor; Ext: extensor.

Before the C5 segmental hemisection, the motion threshold (1 T) of the rats for the preliminary experiment was 110.5 ± 70.5 *μ*A. In response to the stimulation of the right pyramid (at the optimum stimulation intensity of 2 T), all the rats in this group exhibited electric potential on the left side. The average CMAP amplitude of the left forelimb flexor was 780 ± 709 *μ*V and its average latency 8.3 ± 0.95 ms, and the average CMAP amplitude of the left forelimb extensor was 1259 ± 1000 *μ*V and its average latency 7.86 ± 0.82 ms. However, neither the flexor nor the extensor on the right side exhibited CMAPs. Likewise, in response to the stimulation of the left pyramid, all the rats exhibited electric potential on the right side. The average CMAP amplitude of the right forelimb flexor was 516 ± 671 *μ*V and its average latency 8.1 ± 0.83 ms, and the average CMAP amplitude of the right forelimb extensor was 1238 ± 1191 *μ*V and its average latency 7.76 ± 0.43 ms. Neither the flexor nor the extensor on the left side exhibited CMAPs (Figures 5(a) and 5(b)).

**Figure 6:**
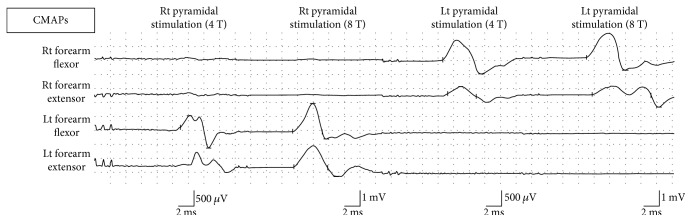
CMAPs obtained by stimulating the pyramid of the rats for preliminary experiment. Despite the increase in stimulation intensity from 4 T to 8 T for both the left and right pyramids, no ipsilateral CMAPs were recorded.

Then, with the right pyramidal stimulation, the stimulation intensity was increased to 4 T (480 *μ*A) and then to 8 T (960 *μ*A), but no CMAPs were recorded on the stimulated side. Likewise, with the left pyramidal stimulation, the stimulation intensity was increased to 4 T (480 *μ*A) and then to 8 T (960 *μ*A), but no CMAPs were recorded on the stimulated side (Figure 6).

**Figure 7:**
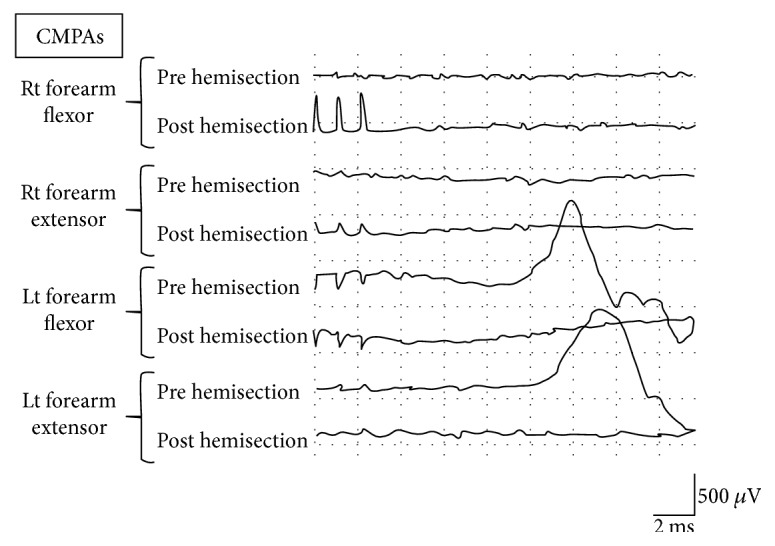
CMAPs obtained by stimulating the right pyramid of the rats for preliminary experiment having undergone left C5 segmental hemisection (stimulation intensity: 2 T). Upper row: before hemisection; lower row: after hemisection. No electric potential of the left forelimb flexor/extensor was recorded immediately after the hemisection.

Immediately after the left segmental hemisection, in response to the right pyramidal stimulation, the CMAPs of the left forelimb flexor and that of the extensor were lost (Figure 7).

### 3.2. Rats for Hemisection

All 6 rats used for the study of functional recovery at 1 to 2 weeks after the surgery exhibited CMAPs of the contralateral forelimb flexor and extensor in response to the right pyramidal stimulation, and two-thirds of them also exhibited CMAPs of the ipsilateral forelimb flexor and extensor. Likewise, in response to the left pyramidal stimulation, all 6 rats exhibited CMAPs of the contralateral forelimb flexor and extensor, and two-thirds of them exhibited CMAPs of the ipsilateral forelimb flexor and extensor. Furthermore, all 6 rats used for the study of functional recovery at 4 to 6 weeks after the surgery exhibited CMAPs on both the right and left sides in response to the right or left pyramidal stimulation (Table 1).

In response to the right pyramidal stimulation, the group of 12 rats that received an additional right C2 segmental hemisection exhibited the following: an average CMAP amplitude of the left forelimb flexor of 496 ± 784 *μ*V and average latency 6.0 ± 1.02 ms; an average CMAP amplitude of the extensor of 296 ± 207 *μ*V and average latency 6.04 ± 0.95 ms; an average CMAP amplitude of the right forelimb flexor of 420 ± 226 *μ*V and average latency 5.85 ± 1.07 ms; and an average CMAP amplitude of the right forelimb extensor of 536 ± 391 *μ*V and average latency 5.85 ± 1.07 ms. Also, in response to the left pyramidal stimulation, the group exhibited the following: an average CMAP amplitude of the right forelimb flexor of 498 ± 333 *μ*V and average latency 6.42 ± 1.14 ms; an average CMAP amplitude of the right forelimb extensor of 526 ± 350 *μ*V and average latency 6.28 ± 1.29 ms; an average CMAP amplitude of the left forelimb flexor of 580 ± 581 *μ*V and average latency 6.29 ± 1.07 ms; and an average CMAP amplitude of the left forelimb extensor of 569 ± 679 *μ*V and average latency 6.48 ± 1.38 ms (Figures 5(a) and 5(b)).

Comparison of the CMAP amplitude and latency between the group of rats for the preliminary experiment and the group that received the additional C2 segmental hemisection exhibited no significant difference in amplitude, but a significant shortening of latency was found with the group that received the additional C2 segmental hemisection (^∗^*p* < 0.05) (Figures 5(a) and 5(b)).

**Figure 8:**
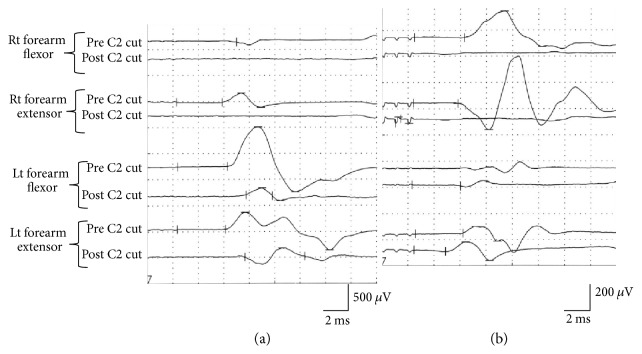
CMAPs (stimulation intensity: 2 T) of rats having undergone additional right C2 hemisection. (a) Immediately after right C2 hemisection, electric potential of the right forelimb was lost in response to the stimulation of the right pyramid, whereas the electric potential of the left forelimb was not lost although the waveform amplitude decreased. (b) Immediately after right C2 hemisection, electric potential of the right forelimb was lost in response to the stimulation of the left pyramid, whereas the electric potential of the left forelimb was not lost although the waveform amplitude decreased.

**Figure 9:**
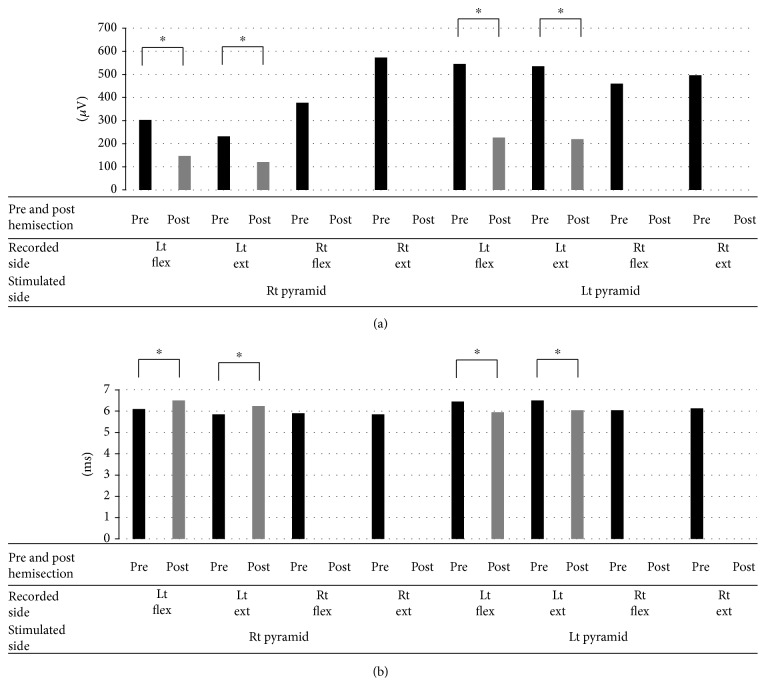
Amplitude (a) and latency (b) of CMAPs before and after the additional C2 hemisection performed on the C2 rat group. (a) In response to the stimulation of both the left and right pyramids, CMAPs of the right forelimb were lost after the additional right C2 hemisection, whereas the amplitude of CMAPs of the left forelimb was not lost although it decreased significantly (^∗^*p* < 0.05). (b) Significant extension of the latency was found in the record of the left forelimb in response to the stimulation of the right pyramid, whereas a significant shortening of latency was found in the record of the left forelimb in response to the stimulation of the left pyramid (^∗^*p* < 0.05). CMAPs: compound muscle action potentials; Rt: right; Lt: left; Flex: flexor; Ext: extensor.

With the group that received the additional C2 segmental hemisection, their right pyramid was stimulated, and then a right C2 segmental hemisection was performed. The average CMAP amplitude of their right forelimb flexor, which was 377 ± 195 *μ*V on average before the surgery, changed to 0 *μ*V, and the CMAP amplitude of their right forelimb extensor, which was 573 ± 391 *μ*V on average before the surgery, was also lost. Meanwhile, the average CMAP amplitude of their left forelimb flexor decreased significantly from 303 ± 254 to 147 ± 94 *μ*V and that of their left forelimb extensor also exhibited a significant decrease from 232 ± 178 to 121 ± 77 *μ*V, but was not lost (*p* < 0.05) (Figures 8(a) and 9(a)). As the result of the left pyramidal stimulation, the average CMAP amplitude of their right forelimb flexor, which was 460 ± 316 *μ*V before the surgery, was lost (0 *μ*V) and also that of their extensor, which was 496 ± 332 *μ*V, was lost (0 *μ*V). Whereas a significant decrease was found in the average CMAP amplitude of their left forelimb flexor, from 545 ± 622 to 227 ± 183 *μ*V, and also in that of their extensor, from 535 ± 721 to 220 ± 219 *μ*V, it was not lost (^∗^*p* < 0.05) (Figures 8(b) and 9(a)). Significant extension of latency was found in the left forelimb record as the result of the right pyramidal stimulation (^∗^*p* < 0.05), and significant shortening was found in the left forelimb record as the result of the left pyramidal stimulation (^∗^*p* < 0.05) (Figure 9(b)).

**Figure 10:**
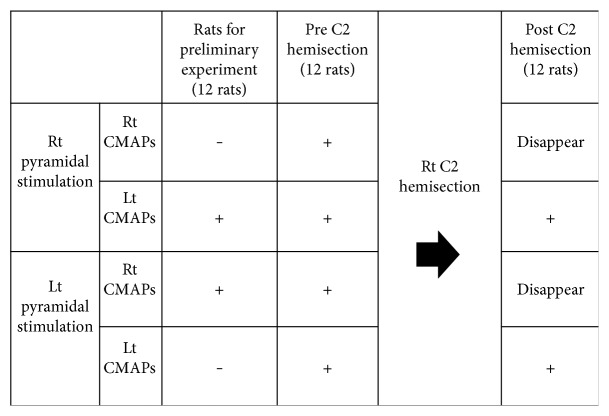
Summary of the results of this experiment. All the rats in the group for the preliminary experiment (those that later received the left C5 segmental hemisection) exhibited contralateral electrical activity in response to the stimulation on one side. Also, at 6 weeks after the C5 hemisection surgery, all 12 rats exhibited CMAPs from both forelimb muscles in response to the stimulation on one side before the additional C2 hemisection. With the rats that received the additional C2 hemisection, only the electric potential on the right side was lost. CAMPs: compound muscle action potentials; Rt: right; Lt: left.

The results are summarized as follows: all the rats in the group for the preliminary experiment exhibited contralateral CMAPs only as the result of stimulation of the other side before the surgery. All 6 rats used for the study of functional recovery at 4 weeks after the hemisection onward exhibited CMAPs of both forelimb muscles as the result of stimulation of one side. Although no change was found in the amplitude, latency was found to have decreased. As the result of the additional right segmental hemisection at C2 level, which is closer to the head, only the electric potential on the hemisected side was lost (Figure 10).

## 4. Discussion

In the development process of the corticospinal tract of the rats, the leading axons are at the level of the medulla oblongata immediately after birth and extend toward the lumbosacral cord over a period of approximately 3 weeks, reaching the state of adult rats [9, 10]. Corticofugal projection fibers also form axonal projections on the neurons existing in the red nuclei, vestibular nuclei, bulbar ventral reticular nuclei, and the bulbar raphe nuclei of the brainstem, which is considered to be involved in motor control [11–13]. It has been confirmed using adult rats that unilateral damage in the cerebral hemisphere caused during the juvenile period (when corticospinal tract axons are still forming projections in a caudal direction) allows the corticofugal projection fibers that are descending from the sensorimotor area of the cerebral cortex on the undamaged side to form bilateral axonal projections at each level of the thalamus [14]; striatum [15]; superior colliculus, red nuclei, and pontine nuclei in the midbrain [16]; pyramidal decussation [17]; and spinal cord [18]. We confirmed this bilateral axonal projection by electrophysiological verification [19]. Past studies have reported cases of cervical spinal cord contusion injury and simple selective cutting [20–23], but there have been no reports on a case where segmental cervical cord hemisection was performed to eliminate the possibility of readhesion of the nerve fibers in the hemisected area. The present study adopted this injury method because it allows assessment of the reproducible compensation function by clearly leaving the intact part. Also, regarding electrophysiological evaluation, there have been no reports on a case where the pyramid of one side is selectively subjected to microstimulation to assess functional recovery over time.

The result of electrophysiological assessment of the corticospinal tract of adult rats, which was the first objective of this study, was as follows: only contralateral forelimb compound muscle action potentials (CMAPs) were recorded in response to the stimulation of the pyramid on one side, and no ipsilateral CMAPs were recorded even with stimulation of an intensity 8 times as high as the exercise threshold. The corticospinal tract of the rats originates from the pyramidal neuron of layer V of the sensorimotor cortex of the brain, and the axons descend along the internal capsule, cerebral peduncle, and the pyramid of the ventral medulla oblongata. Axonal projections are formed along two pathways: 90 to 95% of the axons cross the pyramid, extending toward the dorsal funiculus on the opposite side, and the remaining axons extend toward the ventral spinal cord on the same side without crossing the pyramid [24, 25]. The existence of such ipsilateral ventral-descending neural circuit was not confirmed by the electrophysiological assessment of the corticospinal tract of adult rats conducted this time.

Regarding the time of recovery from motor paralysis in the electrophysiological evaluation, which was the second objective of this study, all 12 rats exhibited CMAPs from both the forelimb muscles in response to the stimulation on one side at 4 weeks after the surgery onward. This result was consistent with the results of recovery time in our behavioral assessment [3]. The past reports also indicated similar results: recovery in 4 to 6 weeks [26, 27].

By comparing the CMAP waveform of the rats for the preliminary experiments and that of the rats for the additional hemisection, shortening in latency was observed in the latter (Figure 6, Table 1). Furthermore, as a result of performing the additional right C2 segmental hemisection to confirm the existence of compensation pathways, which was the last objective of the present study, the electric potential on the right side was lost, and a decrease in electric potential was found on the left side. There are various reports on the study of compensation pathways formed after an injury in the central nervous system of rodents. Also, with rodents, even if half of their thoracic cord is cut off, they do not lose their walking ability, and the involvement of serotonin (5-hydroxytryptamine (5-HT)) fibers in this compensation mechanism has been reported [28–30]. In this model, the mechanism of activating motor cells in the circuit for adjusting walking motion patterns (central pattern generator (CPG)) [31–33] and budding of 5-HT fibers at the lumbar cord level after thoracic cord hemisection [29, 30] has been confirmed.

With adult rats that received dissection of the pyramid on one side and were administered mAb, sprouting from the healthy side toward the damaged side was confirmed at the brainstem and spinal cord levels after the functional recovery [34]. Also, with rats whose dorsal funiculi on both sides were cut off completely at a high C3 level, it has been reported that their motor function was restored naturally in approximately 4 weeks; sprouting from the undamaged ventral corticospinal tract was confirmed; and furthermore, when the ventral corticospinal tract was cut, recovery from paralysis was not observed [33]. As described above, also with adult rodents, sprouting across the damaged and healthy sides and that from the ventral corticospinal tract has been confirmed. Meanwhile, as compensation of the forelimb motor function different from these recovery pathways, C3-C4 propriospinal neurons were reported for the first time with cats. They form an indirect corticomotoneuronal pathway together with a direct pathway for direct projection from the brain to the motor neurons [35–38]. The propriospinal neurons have been reported to exist also in primates and rats, and they are considered extremely important for the recovery of the motor function of fingers in particular [39–42]. In this study, the left propriospinal neurons were cut by the C5 segmental hemisection of the left cervical cord. Meanwhile, since the right propriospinal neurons remained intact, there is a possibility that they were involved in the recovery. However, since the corticospinal tracts on the left and right sides were cut at different levels, the compensation pathways cannot be accounted for without the sprouting across the damaged and healthy sides. Although the extension of latency was thus expected, the opposite result was obtained due to causes such as anesthetic depth and difference in nerve conduction velocity between the dorsal and ventral sides of the corticospinal tract. Technical factors may also have been involved. To identify the exact causes, we consider it necessary to conduct anatomical verification by neuroanatomical tract tracing.

## 5. Conclusion

**Figure 11:**
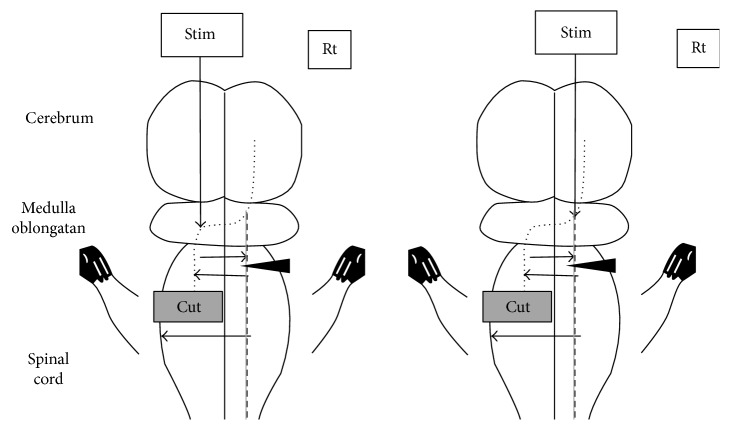
Compensation pathway of the corticospinal tract after the recovery of motor function. Stim: stimulation; Rt: right; cut: left C5 segmental hemisection of cervical cord; short broken line: dorsal corticospinal tract (posterior funiculus); long broken line: ventral corticospinal tract; arrow: sprouting; gray line: activation of the ventral corticospinal tract; arrow head: contralateral additional C2 hemisection.

These results suggest that sprouting across the damaged and healthy sides, activation of the ventral corticospinal tracts, and propriospinal neurons were complexly involved in the growth of compensation pathways assumed by the electrophysiological verification (Figure 11).

## Figures and Tables

**Table 1:** Existence or nonexistence of CMAPs in the group of rats for preliminary experiment and the group of rats for assessment over time after C5 hemisection.

Stimulated side	Recorded side	Rats for preliminary experiment (15 rats)	Assessment over time after C5 hemisection			
			Post hemisection 1 week (3 rats)	Post hemisection 2 weeks (3 rats)	Post hemisection 4 weeks (3 rats)	Post hemisection 6 weeks (3 rats)
Rt pyramid	Rt flex	0/15	2/3	2/3	3/3	3/3
	Rt ext	0/15	2/3	2/3	3/3	3/3
	Lt flex	15/15	3/3	3/3	3/3	3/3
	Lt ext	15/15	3/3	3/3	3/3	3/3

Lt pyramid	Rt flex	0/15	3/3	3/3	3/3	3/3
	Rt ext	0/15	3/3	3/3	3/3	3/3
	Lt flex	15/15	2/3	2/3	3/3	3/3
	Lt ext	15/15	2/3	2/3	3/3	3/3

Number of rats that exhibited electric potential/number of rats measured. All the rats in the group for the preliminary experiment exhibited contralateral electrical potential only in response to stimulation on the opposite side, whereas all the rats assessed over time after the surgery exhibited electric potential on both sides in response to stimulation of the right or left pyramid at 4 to 6 weeks after the surgery. Rt: right; Lt: left; Flex: flexor; Ext: extensor.
