# Corrigendum to “Mechanism of Restoration of Forelimb Motor Function after Cervical Spinal Cord Hemisection in Rats: Electrophysiological Verification”

**DOI:** 10.1155/2018/5627890

**Published:** 2018-07-18

**Authors:** Takumi Takeuchi, Masahito Takahashi, Kazuhiko Satomi, Hideaki Ohne, Atsushi Hasegawa, Shunsuke Sato, Shoichi Ichimura

**Affiliations:** ^1^Department of Orthopaedic Surgery, Kyorin University, 6-20-2 Shinkawa, Mitaka-shi, Tokyo 181-0004, Japan; ^2^Department of Orthopaedic Surgery, Kugayama Hospital, 2-14-20 Kitakarasuyama, Setagaya-ku, Tokyo 157-0061, Japan

In the article titled “Mechanism of Restoration of Forelimb Motor Function after Cervical Spinal Cord Hemisection in Rats: Electrophysiological Verification” [1], there were errors in the compound muscle action potential (CMAP) results reported in the Rats for Hemisection section, Table 1, and Figure 9, as follows:

1. The CMAP amplitudes reported in the fourth paragraph of the Rats for Hemisection section were incorrect. The corrected paragraph is as follows:

“With the group that received the additional C2 segmental hemisection, their right pyramid was stimulated, and then a right C2 segmental hemisection was performed. The average CMAP amplitude of their right forelimb flexor, which was 420 ± 226 *μ*V on average before the surgery, changed to 0 *μ*V, and the CMAP amplitude of their right forelimb extensor, which was 536 ± 391 *μ*V on average before the surgery, was also lost. Meanwhile, the average CMAP amplitude of their left forelimb flexor decreased significantly from 496 ± 784 to 147 ± 94 *μ*V and that of their left forelimb extensor also exhibited a significant decrease from 296 ± 207 to 121 ± 77 *μ*V but was not lost (*p* < 0.05) (Figures 8(a) and 9(a)). As the result of the left pyramidal stimulation, the average CMAP amplitude of their right forelimb flexor, which was 498 ± 333 *μ*V before the surgery, was lost (0 *μ*V) and also that of their extensor, which was 526 ± 350 *μ*V, was also lost (0 *μ*V). Whereas a significant decrease was found in the average CMAP amplitude of their left forelimb flexor, from 580 ± 581 to 227 ± 183 *μ*V, and also in that of their extensor, from 596 ± 679 to 220 ± 219 *μ*V, it was not lost (^∗^*p* < 0.05) (Figures 8(b) and 9(a)). Significant extension of latency was found in the left forelimb record as the result of the right pyramidal stimulation (^∗^*p* < 0.05), and significant shortening was found in the left forelimb record as the result of the left pyramidal stimulation (^∗^*p* < 0.05) (Figure 9(b)).”

2. There were errors in the values in the third column of Table 1. The corrected table is as follows:

3. There were errors in Figure 9(a). The corrected figure is as follows:

## Figures and Tables

**Figure 1:**
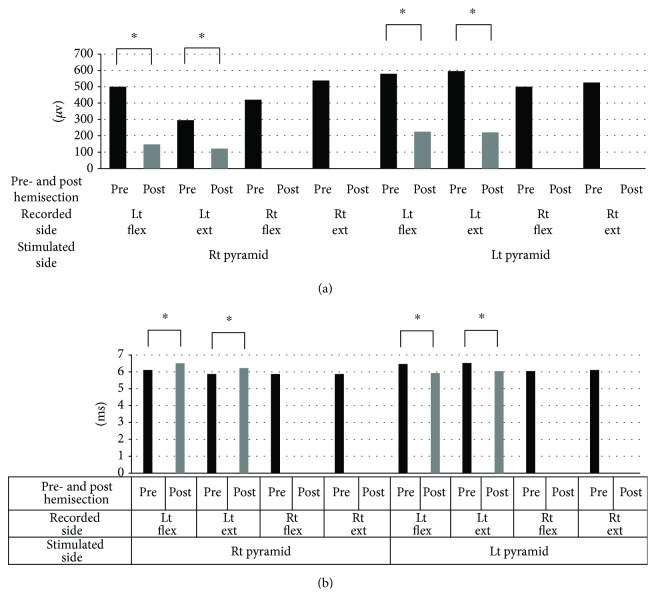
Amplitude (a) and latency (b) of CMAPs before and after the additional C2 hemisection performed on the C2 rat group. (a) In response to the stimulation of both the left and right pyramids, CMAPs of the right forelimb were lost after the additional right C2 hemisection, whereas the amplitude of CMAPs of the left forelimb was not lost although it decreased significantly (^∗^*p* < 0.05). (b) Significant extension of the latency was found in the record of the left forelimb in response to the stimulation of the right pyramid, whereas a significant shortening of latency was found in the record of the left forelimb in response to the stimulation of the left pyramid (^∗^*p* < 0.05). CMAPs: compound muscle action potentials; Rt: right; Lt: left; Flex: flexor; Ext: extensor.

**Table 1:** Existence or nonexistence of CMAPs in the group of rats for preliminary experiment and the group of rats for assessment over time after C5 hemisection.

Stimulated side	Recorded side	Rats for preliminary experiment (15 rats)	Assessment over time after C5 hemisection			
			Posthemisection 1 week (3 rats)	Posthemisection 2 weeks (3 rats)	Posthemisection 4 weeks (3 rats)	Posthemisection 6 weeks (3 rats)
Rt pyramid	Rt flex	0/15	2/3	2/3	3/3	3/3
	Rt ext	0/15	2/3	2/3	3/3	3/3
	Lt flex	15/15	3/3	3/3	3/3	3/3
	Lt ext	15/15	3/3	3/3	3/3	3/3

Lt pyramid	Rt flex	15/15	3/3	3/3	3/3	3/3
	Rt ext	15/15	3/3	3/3	3/3	3/3
	Lt flex	0/15	2/3	2/3	3/3	3/3
	Lt ext	0/15	2/3	2/3	3/3	3/3
